# Species Quantification in Complex Herbal Formulas—Vector Control Quantitative Analysis as a New Method

**DOI:** 10.3389/fphar.2020.488193

**Published:** 2020-11-26

**Authors:** Bo Zhao, Chao Xiong, Jingjian Li, Deng Zhang, Yancai Shi, Wei Sun, Xiaoqun Duan

**Affiliations:** ^1^ College of Pharmacy, Guilin Medical University, Guilin, China; ^2^ Institute of Chinese Materia Medica, China Academy of Chinese Medical Sciences, Beijing, China; ^3^ School of Biology and Pharmaceutical Engineering, Wuhan Polytechnic University, Wuhan, China; ^4^ Guangxi Institute of Botany, The Chinese Academy of Sciences, Guilin, China

**Keywords:** complex herbal formulas, vector control quantitative analysis, internal transcribed spacer region, species quantification, limit of quantification, traditional Chinese medicine quality control

## Abstract

Product mislabeling and/or species fraud in Traditional Chinese Medicine (TCM) not only decrease TCM quality, but also pose a potential health issue to the end user. Up to now, methods to control TCM quality have been developed to detect specific metabolites or identify the original species. However, species quantification in complex herbal formulas is rarely concerned. Here, we reported a simple Vector Control Quantitative Analysis (VCQA) method for flexible and accurate multiplex species quantification in traditional Chinese herbal formulas. We developed PCR-based strategy to quickly generate the integrated DNA fragments from multiple targeted species, which can be assembled into the quantitative vector in one round of cloning by Golden Gate ligation and Gateway recombination technique. With this method, we recruited the nuclear ribosomal DNA Internal Transcribed Spacer (ITS) region for the quantification of *Ligusticum sinense* “Chuanxiong,” *Angelica dahurica* (Hoffm.) Benth. & Hook.f. ex Franch. & Sav., *Notopterygium incisum* K. C. Ting ex H. T. Chang, *Asarum sieboldii* Miq., *Saposhnikovia divaricata* (Turcz.) Schischk., *Nepeta cataria* L., *Mentha canadensis* L., and *Glycyrrhiza uralensis* Fisch. ex DC. in ChuanXiong ChaTiao Wan, a classic Chinese herbal formula with very long historical background. We found that, firstly, VCQA method could eliminate the factors affecting such as the variations in DNA extracts when in combination with the use of universal and species-specific primers. Secondly, this method detected the limit of quantification of *A. sieboldii* Miq. in formula products down to 1%. Thirdly, the stability of quality of ChuanXiong ChaTiao Wan formula varies significantly among different manufacturers. In conclusion, VCQA method has the potential power and can be used as an alternative method for species quantification of complex TCM formulas.

## Introduction

Traditional Chinese Medicine (TCM) is the key element of traditional Chinese medical system and its culture and civilization have evolved over thousands of years. Despite the advanced medical technologies in recent years, TCM is still playing an important role in primary healthcare for Chinese people and even worldwide. Indeed, many herbal remedies of TCM have made it into modern medicines through drug development and their use continues to increase. China possesses a diverse and rich flora to form the basis of traditional medicine, and more than 3,200 herbs are used in different formulas (Daxue Consulting, 2015). Just in the Pharmacopoeia of the People’s Republic of China, more than 500 herbal plants and their extracts and almost 300 complex herbal formulations are recorded (State Pharmacopoeia Committee, 2015), not to mention the other ethnic pharmacopoeias in China. Herbal formula usually contains multi-ingredients from two or more plant species and has the competence to systematically heal illness. Different to those herbal medicines that contain only one herb, the complex formula uses one or two main ingredients as pioneers to target and treat the core symptom, while the other ingredients are used to treat other symptoms caused by the disease, helping the main herbs to enhance their positive effects and finally eliminate the signs and symptoms of disease exactly (Wang et al., 2008; Daxue Consulting, 2015). Owing to this excellent characteristic, it is not surprising that herbal formulas have become more and more popular and attract consumers’ preferences around the world. A statistics from hexa research reported that the global herbal medicine market value reached USD 71.19 billion in 2016 and is expected to exhibit profitable growth over the forecast period (Hexa Research, 2017). In addition, the rapid industrialization and modernization also pose profound effects on herbal medicine supply (e.g., expanding the commercialization of herbal medicinal products throughout various markets, including the Internet) (Li et al., 2016a; Xiong et al., 2018). In this context, quality control of herbal medicine products is a crucial part that cannot be overlooked. Although the Pharmacopoeia has strict species dosage listed for herbal formulas and recommend the manufacturer to produce accordingly, no stringent stipulations of quality control have been established. This situation leaves loopholes for some unscrupulous manufacturers who tend to use fake herb species or incorrectly label species percentage in the products (Chen et al., 2014; Osathanunkul et al., 2015a; Li et al., 2016b). Any case of herbal medicine fraud not only decreases product quality but also causes potential health issue to the end user.

**FIGURE 1:**
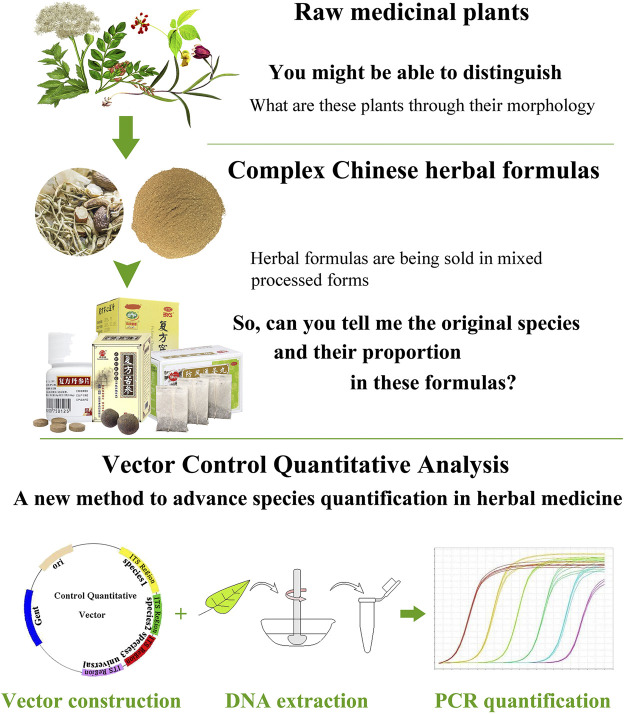
VCQA method shows a potential power in species quantifying for herbal medicine formulas.

To date, methods to control herbal medicine quality have been developed, involving morphological and microscopic identification and chemical constituents’ analysis. These classical methods could identify plant species in herbal medicine product based on morphological characteristics or determine specific chemical components based on related reference substance (Franz et al., 2007; Liu et al., 2017). Remarkably, several new reviews highlighted the efficiency and increasing application of DNA-based methods in herbal medicine identification (Sucher and Carles, 2008; Chen et al., 2014; Li et al., 2016a). From their reviews, we notice that DNA barcoding methods which are originally developed to identify organisms have been widely adopted for the identification of single species in herb product (Techen et al., 2014; Mishra et al., 2016). However, due to the shortcoming of Sanger sequencing, current DNA barcoding methods are not sufficient to simultaneously identify multispecies in complex medicinal preparations (i.e., herbal formulas). In the Chinese Pharmacopoeia, herbal formulas are formulated with two or more species in prescribed proportions and are processed into different dosage forms such as tablets, capsules, powders, extracts, pastes, gels, and oils (Figure 1). It is almost impossible to identify certain biological ingredients in herbal formulas through Sanger sequencing. In addition, the present DNA barcoding methods are emphasizing on species authentication of herbal medicine, yet little concern has been focused on the species quantification in herbal formulas, which we believe to be an important yet underappreciated factor in quality control. As we know, some herbal formulas contain toxic species (e.g., ChuanXiong ChaTiao Wan contains *Asarum sieboldii* Miq.; ShuFeng DingTong Wan contains *Strychnos nux*-*vomica* L.; YuZhen San contains *Arisaema heterophyllum* Blume; LiuWei MuXiang San contains *Rhododendron molle* (Blume) G.Don). Excess toxic ingredients in formula may lead to chronic injury on the human body (Wu et al., 2015). In principle, DNA quantification methods target either low copy genes in nuclear genome or single copy genes in plastid DNA. However, contrary to nuclear DNA, the quantity of ptDNA exhibits high variation among different tissue; hence, quantification cannot be based on ptDNA; instead a low copy nuclear DNA target is appropriate for herbal formulas. The Internal Transcribed Spacer (ITS) sequence has been used as a core barcode for plant identification and classification because this locus has high mutation rate (Chen et al., 2010; Li et al., 2011). According to these studies, we hypothesis that ITS region may fulfill the requirements for the species quantification of complex herbal formulas.

In the past, most of the studies preferred to use relative quantitative real-time PCR to quantify meat species in food; however, this method is affected by many factors (Laube et al., 2007). One such factor is the unavailability of an exogenous standard robust enough for use in a complex product like sample mixed with different type tissue. In absolute quantification, although some studies used PCR or restriction digestion to perform quantitative analysis of parasite or virus in blood, none of them can simultaneously quantify more than three targets (Workenhe et al., 2008; Kamau et al., 2013; Gotia et al., 2016). In this work, we developed a Vector Control Quantitative Analysis (VCQA) method that enables efficient quantification of multiple species in herbal formula products (Figure 1). VCQA is amenable to high throughput but with reduced costs, it has several advantages over previous quantitative analyses: 1) VCQA uses PCR-based procedures to quickly produce multiple species-specific DNA fragments with designated *Bsa*I enzyme digestion sites at their two ends, which could assemble into a single quantitative vector in one round of cloning by Golden Gate ligation and Gateway recombination. 2) VCQA could eliminate the factors affecting such as the variations in DNA extracts when in combination with the use of universal and species-specific primers. 3) The data produced by VCQA can change into mg/mg for species quantification, which is consistent with the norms described in the Chinese Pharmacopoeia. To elucidate the workflow of quantification of this approach, we described a systematic pipeline for the quantitative determination of ChuanXiong ChaTiao Wan formula in this study.

## Materials and Methods

### Reference Sample and Commercial Products Collection

**FIGURE 2:**
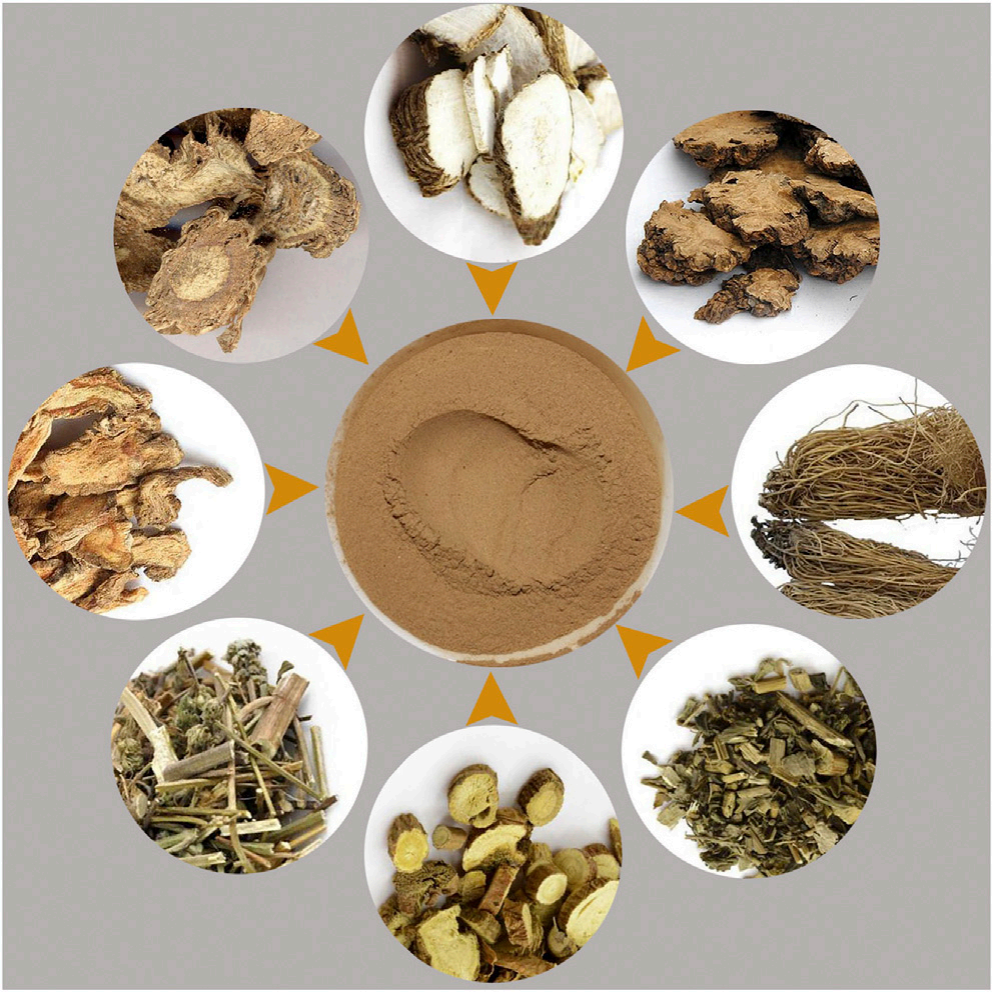
Reference samples of ChuanXiong ChaTiao Wan used for VCQA.

To test the accuracy and repeatability of VCQA method, a lab-made reference ChuanXiong ChaTiao Wan sample was formulated with authenticated herbal materials according to the prescription documented in the Chinese Pharmacopoeia. Eight herbal materials were collected from Tong Ren Tang drug store, including *Ligusticum sinense* 'Chuanxiong', *Angelica dahurica* (Hoffm.) Benth. & Hook.f. ex Franch. & Sav., *Notopterygium incisum* K.C.Ting ex H.T.Chang, *Asarum sieboldii* Miq., *Saposhnikovia divaricata* (Turcz.) Schischk., *Nepeta cataria* L., *Mentha canadensis* L., and *Glycyrrhiza uralensis* Fisch. ex DC. (Table 1). All samples were authenticated using DNA barcoding method according to the protocol described in the Chinese Pharmacopoeia (Chen et al., 2014; State Pharmacopoeia Committee, 2015). All of the corresponding voucher materials were deposited in Guangxi Institute of Botany. The reference ChuanXiong ChaTiao Wan sample was processed as follows: the eight herbal materials were respectively ground into powder, and then the powder was sieved and evenly mixed according to the official proportion (Table 1; Figure 2). To test the sensitivity of VCQA method, an increasing quantity of *Asarum sieboldii* Miq. powder (0.1, 1, 2, 5, and 10%) was added to a standard mixture of ChuanXiong ChaTiao Wan formula which does not contain *Asarum sieboldii* Miq. Three repetitions performed in one run were used for the ChuanXiong ChaTiao Wan mixtures. The limit of quantification is defined as the lowest percentage of *Asarum sieboldii* Miq. that could be stably detected in all qPCRs. To evaluate the practical application capacity of VCQA method, ten commercial products of ChuanXiong ChaTiao formula produced by different manufacturers were purchased from different drug stores for this study.

**TABLE 1:** Sample list of the botanical species in ChuanXiong ChaTiao Wan.

Species name	Weight (g)	Part used	Voucher number
*Ligusticum sinense*	120	Rhizome	RF01LC01∼05
*Angelica dahurica*	60	Root	RF02AD01∼05
*Notopterygium incisum*	60	Rhizome and root	RF03NI01∼05
*Asarum sieboldii*	30	Root and rhizome	RF04AS01∼05
*Saposhnikovia divaricata*	45	Root	RF05SD01∼05
*Nepeta cataria*	120	Stem and leaf	RF06NC01∼05
*Mentha canadensis*	240	Stem and leaf	RF07MC01∼05
*Glycyrrhiza uralensis*	60	Root and rhizome	RF08GU01∼05

### DNA Extraction and Primer Design

Total DNA was extracted according to the protocol of CTAB as modified by Sun (Sun et al., 2016). DNA concentration was quantified using a Qubit 3.0 spectrophotometer (Invitrogen, USA) and adjusted to 50 ng/μl working concentration and stored at −20°C until needed. The ITS region was amplified using universal primers ITS4 and ITS5 (Supplementary Table S1). PCR was performed in a volume of 20 μl containing 10 μl of 2 × Taq PCR mix (Sangon Biotech Co., China), 0.5 μl of DNA template, 1 μl of each primer (final concentration of 0. 25 μM), and 7.5 μl of ultrapure water. The following PCR program was used: 95°C for 3 min; 32 cycles at 95°C for 20 s, 55°C for 20 s, and 72°C for 30 s; and a final extension cycle at 72°C for 5 min. PCR product was sequenced in two directions by Sanger sequencing. Based on the sequencing results, we designed species-specific primers and universal primers for *Ligusticum sinense* “Chuanxiong,” *Angelica dahurica* (Hoffm.) Benth. & Hook.f. ex Franch. & Sav., *Notopterygium incisum* K.C.Ting ex H.T.Chang, *Asarum sieboldii* Miq., *Saposhnikovia divaricata* (Turcz.) Schischk., *Nepeta cataria* L., *Mentha canadensis* L., and *Glycyrrhiza uralensis* Fisch. ex DC., respectively (Supplementary Table S1). The sequence alignment was carried out by MUSCLE in software MEGA 5.0. Regions of species divergence were selected for designing primer to amplify specific product for a particular species, while the universal primer sites were chosen at conserved sites to give a PCR product size of 120 bp.

### Cloning of Multiple Target Sequences in Quantitative Vector

A restriction–ligation–recombination reaction (15 μl) was prepared to contain 1x *Bsa*I reaction buffer (NEB) plus 1.5 μl of 10x NEB ligation buffer (which contains 10 mM ATP), 5 U of *Bsa*I, 20 U of T4 DNA ligase (NEB), and the purified PCR products (10 ng for each species) amplified with the Pps and Pgs primers. The reaction was incubated in a thermocycler for 15 cycles (37°C 2 min, 10°C 3 min, and 20°C 5 min) and then terminated at 65°C for 5 min; when the reaction cooled down to room temperature, 20 ng of pDONR207 plasmid and 3 μl of BP Clonase^™^ buffer (Invitrogen, USA) were added to the reaction and incubated at 25°C for more than 2 h. However, in cases with five or more species-specific fragments and with the reactions not optimally set, the ligation efficiency may be relatively poor. Therefore, as an optional step the ligated products could be used as templates to amplify the linked fragments using two flanking primers (i.e., Pps-LcITS_F and Pgs-GuITS_R, Supplementary Table S1). A PCR (25 μl) with 3.0 μl of the ligated product and 12.5 μl 2x PrimeSTAR^®^ Max DNA Polymerase was set up and thermal cycling was performed with 15 cycles (98°C, 10 s, 58°C, 15 s, and 72°C 3 min). Specific PCR product was purified from agarose gel using a gel purification kit (Tiangen, China), and 20 ng of the PCR product and 20 ng of the pDONR207 plasmid were subjected to Gateway BP recombination as described above.

The reaction products with multiple specific fragments (up to eight for ChuanXiong ChaTiao Wan formula) were directly used to transform *E. coli* competent cells. Positive colonies on an LB-agar medium plate containing 50 mg/L of Gentamycin were selected for further analysis by PCR and sequencing using flanking primer pDONR_F and pDONR_R, and species-specific primers were used as internal primers.

### Preparation of Plasmid DNA Standards

The positive colony was grown up overnight in 10 ml LB liquid cultures, with shaking at 180 rpm at 37°C, and plasmid was subsequently extracted using a plasmid extraction kit (Tiangen, China). In order to generate standard curves, the plasmid was linearized by digesting with EcoR V enzyme (NEB) in a 50 μl reaction volume following the manufacturer’s protocol. The linearized DNA was then purified using the PCR product purification kit (Tiangen, China) and was quantified using a Qubit 3.0 spectrophotometer. With the molecular weight of the plasmid known, it is able to calculate the copy number by the following formula (Kamau et al., 2013): Number of copies/μl = 6.022 × 10^23^ (molecules/mole) × DNA concentrations (g/μl)/number of bases pairs × 660 daltons.

Based on the concentration of plasmid DNA and its copy number, the accurate amount of molecules added to subsequent real-time PCR runs can be computed to establish a standard curve for the quantification of specific DNA (Cao and Luo, 2016).

### Quantification of Specific Species in Herbal Formula Using Vector Control Quantitative Analysis Combined With qPCR

RT-qPCR was performed on LightCycler 480 (Roche) real-time PCR machine. Briefly, the 20 μl reaction containing 10 μl 2x SYBR Premix Ex Taq II Mix, 1 μl diluted DNA template, and 1 μl of each primer (final concentration of 0. 25 μM) was added to each well. Samples were amplified for 40 cycles of 95°C for 15 s, 58°C for 10 s, and 72°C for 20 s. After the last reaction cycle, melting curve analysis was carried out immediately from 55 to 95°C in 0.15°C/s increments to determine the specificity of the RT-PCR products. For construction of the standard curves, tenfold dilution series of linearized plasmid DNA starting from 10^10^ to 10^5^ specific copies/μl were used as DNA template to construct the standard curve. In order to use standard curves to quantify the specific species in ChuanXiong ChaTiao Wan formula, plasmid DNA was run alongside the DNA from single species, reference formula, and commercial samples. A standard curve was drawn by plotting the threshold cycle (Ct) against the natural log of concentration (copies/μl). Ct value was calculated using default settings in the LightCycler series software. The quality of standard curves was judged by the slope of the standard curve and the coefficient of determination (*R* ^2^). According to the slope of each standard curve, the efficiency (*E*) of PCR amplification can be calculated based on the equation *E* =10 ^(−1/slope)^ − 1. Besides, in the reaction of SYBR^®^ Green I RT-qPCR, melting curve analysis was used to check the specificity of the RT-PCR product.

## Results

### Primer Design and Evaluation

In order to establish a robust method for the quantitative detection of plant species in herbal formula, species-specific and universal primers (used as endogenous control) were combined into a single method in this study. The ITS region is a well-established target for plant species discrimination (Fritsch and Cruz, 2012; Yan et al., 2015; Li et al., 2016c). ITS region has a high mutation rate, which provides the degree of sequence variation required for plant species identification. Therefore, sequence divergence in ITS can be selected to design species-specific primers. In addition, the use of universal primers can provide an endogenous control of qPCR-quantitative DNA presented in the mixed sample. The use of such primers to simultaneously detect a universal fragment is very important for quantitative analysis because it allows accounting for possible amplification differences among the sample extracts due to variations in DNA recovery and quality of the extracts as a result of matrix effects and industrial processing (Iwobi et al., 2015). By a comparison of the species-specific versus endogenous control signal obtained from the samples, the inaccuracies caused by the use of different DNA from different batches can be reduced. To evaluate the specificity of the primer pairs for VCQA, both species-specific and universal primers were tested for their selectivity and cross-reactions by the analysis of DNA obtained from the eight individual species of ChuanXiong ChaTiao Wan. Firstly, the PCR amplified products were detected using electrophoresis in 1.5% agarose gel. Only those primer pairs produced the expected amplicon in corresponding species and no nonspecific bands in any other species were selected for further analysis (data not shown). Secondly, in real-time PCR, the specificity of the primers can be evaluated by the melting temperature (Tm) of the amplification products immediately after the last reaction cycle. The Tm refers to the temperature at which 50% of the DNA amplicon is in the single-stranded configuration. It can be determined when an additional procedure of slow heating from 55 to 95°C in 20 min is recruited in the qPCR reaction. During this period, fluorescence intensity decreased rapidly due to the denaturation of amplicons, because SYBR green could not bind to single-stranded DNA. A good primer pair results in a single amplicon, distinguished by generating a single melting curve peak but not in nonspecific products such as nonspecific band and primer-dimers (Supplementary Figure S1).

Finally, as a target site for species-specific quantification of ChuanXiong ChaTiao Wan, 180, 407, 342, 365, 410, 195, 387, and 362 bp fragment were amplified for *Ligusticum sinense* “Chuanxiong,” *Angelica dahurica* (Hoffm.) Benth. & Hook.f. ex Franch. & Sav., *Notopterygium incisum* K. C. Ting ex H. T. Chang, *Asarum sieboldii* Miq., *Saposhnikovia divaricata* (Turcz.) Schischk., *Nepeta cataria* L., *Mentha canadensis* L., and *Glycyrrhiza uralensis* Fisch. ex DC., respectively, while the target site for the endogenous control (which can be amplified using the new designed universal primers for these eight species) consisted of a ∼120 bp DNA fragment.

### Preparation of Quantitative Vector Constructs for Species Quantifying

**FIGURE 3:**
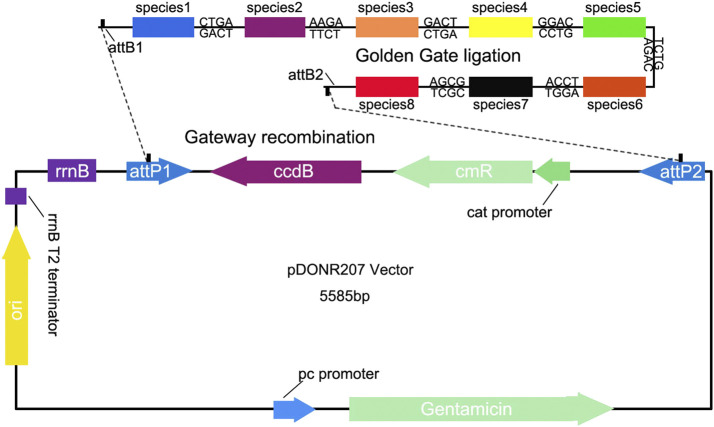
Cloning of eight quantitative fragments into the pDONR207 vectors.

To generate quantitative vector containing the target sequences, firstly we amplified the target fragment using site-specific primers (Pps, Pgs), which include different *Bsa*I-cutting sites at their 5′-end for Golden Gate ligation, and the flanking primers with *att*B sites for Gateway recombination (Supplementary Table S1). Golden Gate ligation uses the special cleavage feature of type IIs restriction endonucleases, such as *Bsa* I, to design and generate distinct, nonpalindromic sticky ends of sequences, which can avoid self-ligation and noncompatible end ligation (Ma et al., 2015). Therefore, this approach is efficient to link multiple DNA fragments in a designed order in a single reaction (Figure 3). Gateway Cloning Technique allows transfer of DNA fragments into a plasmid with two flanking recombination sequences called “attL 1” and “attL 2,” to develop a “Gateway recombination clone.” Using these cloning strategies, we can construct a quantitative vector containing one or multiple species-specific sequences. To test whether this VCQA method can effectively quantify species in herbal formula, we prepared a pDONR207-based construct carrying eight species-specific sequences from the eight target species in ChuanXiong ChaTiao Wan (Figure 3).

### Real-Time PCR System Set Up for Vector Control Quantitative Analysis

To evaluate the applicability of VCQA method for the quantification of plant species in ChuanXiong ChaTiao Wan, both linearized plasmid and standard reference sample were used to construct standard calibration curves based on the parallel amplification of species-specific and/or universal sequences for each target species. Because there are no formal requirements for species quantification of herbal medicine products at present, we recruited some performance criteria of real-time PCR assays for VCQA method. Accordingly, VCQA should be specific to respond exclusively to the target species, which has been comprehensively demonstrated in above section, the standard test including the following criteria according to the document of method performance requirements released by the European Network of GMO Laboratories (ENGL, 2015) with some modification. The coefficient of determination (*R* ^2^) should be above 0.98, and the average value of the slope for the standard curves should be in the range of −3.1 and −3.6, corresponding to amplification efficiencies of 110 to 90%. The limit of quantification refers to the minimum amount of a target species in the product that can be stably quantified within an acceptable level of confidence of 95%, ensuring the false negative results are less than 5%.

**FIGURE 4:**
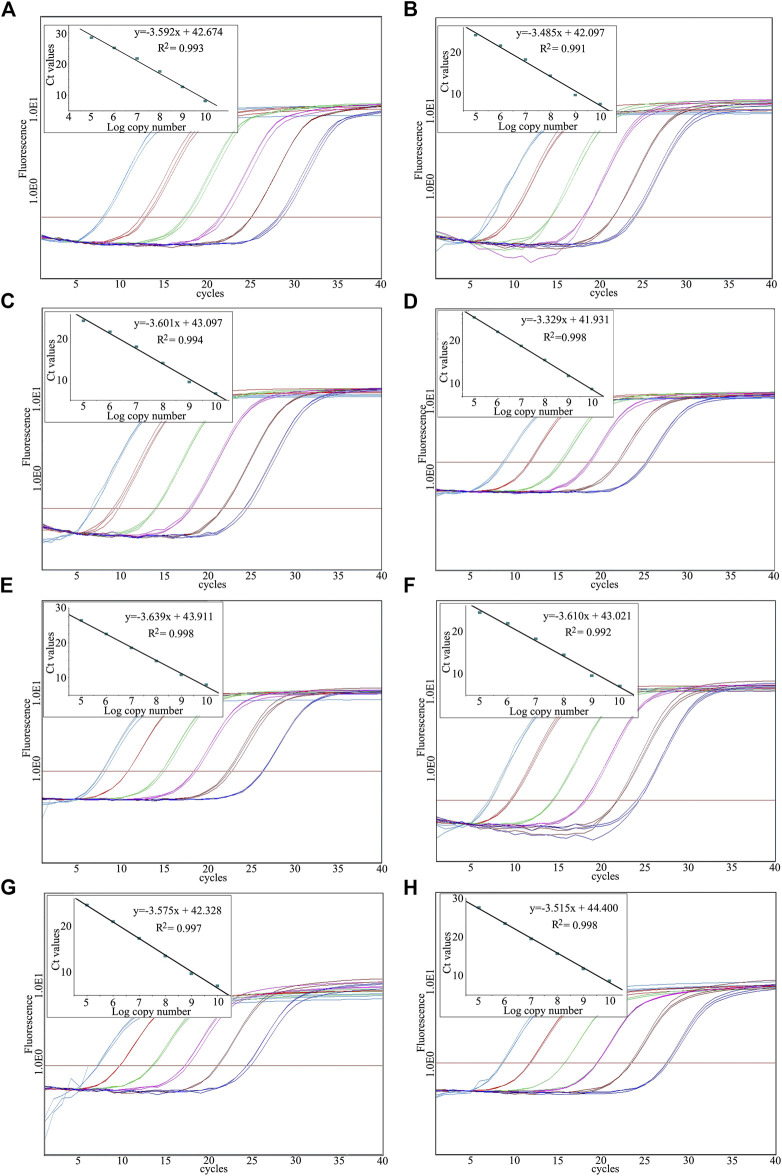
Amplification graph and standard curve constructed using the dilute linearized plasmid. **(A)** *Ligusticum sinense*, **(B)** *Angelica dahurica*, **(C)** *Notopterygium incisum*, **(D)** *Asarum sieboldii*, **(E)** *Saposhnikovia divaricata*, **(F)** *Nepeta cataria*, **(G)** *Mentha canadensis*, **(H)** *Glycyrrhiza uralensis*.

The reliability of standard samples for absolute qPCR is a determining factor. Researchers have paid a lot of effort to develop appropriate standards (e.g., PCR amplified target sequences, plasmids insert with the target sequence) for quantitative analysis (Dhanasekaran et al., 2010). It is accepted that plasmid DNA, especially linearized plasmid, is more reliable for absolute qPCR. Therefore, it is very important to linearize plasmid DNA produced in this study to improve its reliability. After digestion, the absolute qPCR analysis performance of each target sequence could be determined by linearized plasmid. To evaluate the efficiency, we diluted the linearized plasmid DNA 6 times from 10^10^ to 10^5^ specific copies/μl with tenfold serial gradient and analyzed it in 3 replicates (Figure 4). The *R* ^2^ and the slope values of each curve were used to determine the efficiency of each performance, while the standard deviation of each standard calibration curve was employed to determine the assay precision. Consequently, most of the absolute qPCR analysis of the target fragments was performed with efficiency of greater than 90%, *R* ^2^ values were 0.99 or higher, and the standard deviation of each standard calibration curve was lower than 0.7.

In absolute quantification, copy number is used to represent the sample amount. However, for herbal formula, species proportion is commonly expressed as mg/mg according to species dosage as measured by weighing instrument. When performing absolute qPCR for herbal formula product, it is important that species proportion should be expressed as herbal medicine industry norms. Here, a purpose was laid out to determine the amount of copy numbers that is equivalent to mg/mg. The Ct values generated from absolute and relative qPCR assays were correlated to calculate the amount of copy numbers that is equivalent to mg/mg. To determine the species proportion in ChuanXiong ChaTiao Wan, reference samples with known species weight were made. All the eight plant species were detected by real-time PCR analyses using serially diluted plasmid DNA (for absolute qPCR) and genomic DNA (for relative qPCR). The related calibration curves were generated by plotting the calculated Ct value against the logarithm of sample weight. When the two obtained curves exhibited the same linear ranges, we can say that the Ct levels for both curves were equivalent. Therefore, to calculate the amount of copy numbers that is equivalent to mg/mg from the relative qPCR assay, the Ct values obtained from relative qPCR assays were interpolated as unknown copy numbers from the linear regression standard curve of the absolute qPCR assays to obtain equivalent mg/mg. The amount of copy numbers that is equivalent to species weight in mg was calculated according to the averages obtained from series dilutions for qPCR analyses. For the *Ligusticum sinense* “Chuanxiong” absolute qPCR assay, 1208.5 copy numbers corresponds to 1 mg; for the *Angelica dahurica* (Hoffm.) Benth. & Hook.f. ex Franch. & Sav. absolute qPCR assay, 1355.7 copy numbers correlates to 1 mg; for the *Notopterygium incisum* K. C. Ting ex H. T. Chang absolute qPCR assay, 3275.5 copy numbers corresponds to 1 mg; for the *Asarum sieboldii* Miq. absolute qPCR assay, 1664.3 copy numbers correlates to 1 mg; for the *Saposhnikovia divaricata* (Turcz.) Schischk. absolute qPCR assay, 2389.1 copy numbers correlates to 1 mg; for the *Nepeta cataria* L. absolute qPCR assay, 1214.9 copy numbers correlates to 1 mg; for the *Mentha canadensis* L. absolute qPCR assay, 4661.6 copy numbers correlates to 1 mg; for the *Glycyrrhiza uralensis* Fisch. ex DC. absolute qPCR assay, 1338.7 copy numbers correlates to 1 mg.

The estimated weight of each species in the reference formula sample was listed in Table 2. The coefficients of variation corresponding to the repeatability of results obtained under experiment conditions varied from 2.0 to 18.6%, indicating the accuracy of VCQA over the tested dynamic range (<25%). The measure of trueness (i.e., bias) is within ± 25% of the accepted reference value over the whole dynamic range, which demonstrates a close proximity between the estimated and real values (ENGL, 2015). The closeness of agreement between estimated and real values suggests that the developed VCQA method can competently be applied to calculate the species proportion in complex herbal formula products.

**TABLE 2:** Results of reference formulas for the validation of VCQA method.

Species name	Species weight (mg/100 mg)		SD	CV (%)	Bias
	Actual	VCQA predicted			
*Ligusticum sinense*	16.33	14.19	2.83	18.6	−13.1
*Angelica dahurica*	8.16	7.71	1.26	16.3	−5.5
*Notopterygium incisum*	8.16	7.04	0.71	8.8	−12.5
*Asarum sieboldii*	4.08	4.00	0.09	2.3	−1.9
*Saposhnikovia divaricata*	6.12	6.68	0.13	2.0	9.1
*Nepeta cataria*	16.33	14.97	1.75	11.7	−8.3
*Mentha canadensis*	32.65	31.75	2.28	7.2	−2.7
*Glycyrrhiza uralensis*	8.16	8.53	0.91	10.9	4.5

Note: SD, standard deviation. CV, coefficient of variation = (SD/mean) * 100%. Bias = ((mean value-true value)/true value * 100).

**FIGURE 5:**
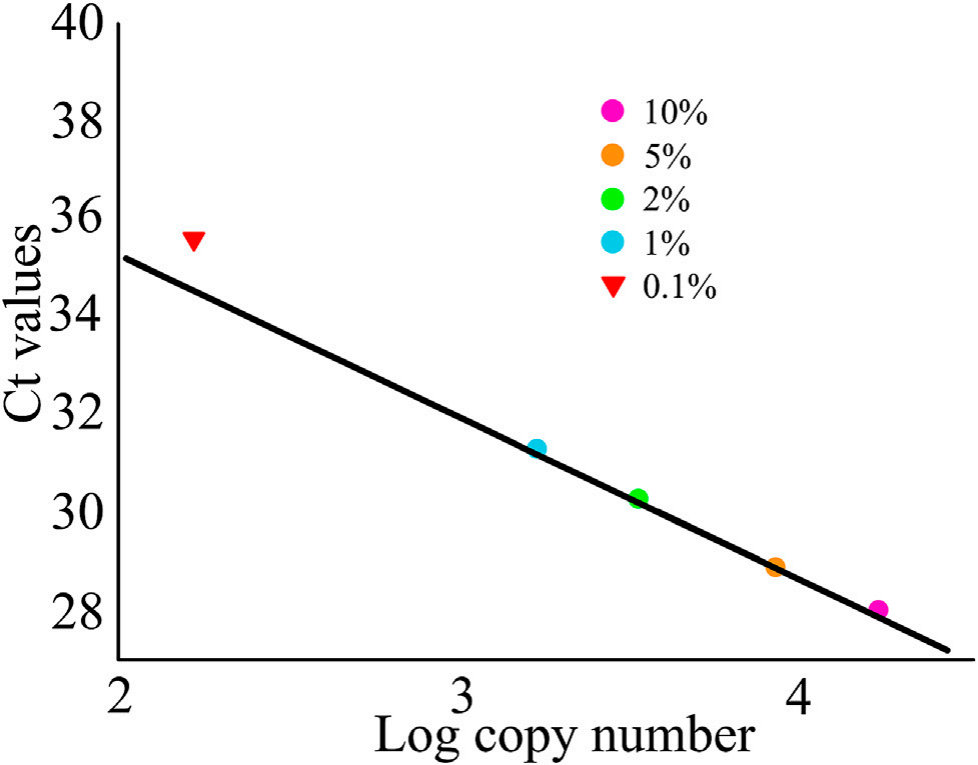
Linear regression plots for the limit quantification of *Asarum sieboldii* in the reference model mixtures of ChuanXiong ChaTiao Wan.

The absolute quantification limit of VCQA method was determined using an increasing quantity of *Asarum sieboldii* Miq. (0.1, 1, 2, 5, and 10%) in standard mixture of ChuanXiong ChaTiao Wan formula. The experimental data showed that *Asarum sieboldii* Miq. could be reliably quantified to 1% level. The results exhibited high performance in terms of linearity (*R* ^2^ = 0.998) and PCR efficiency (98.5%). In addition, we noticed that linearity was vulnerable under the lowest percentage (0.1%) of *Asarum sieboldii* Miq. in formula mixtures (Figure 5). When the level of 0.1% (w/w) was included, PCR efficiency did not comply with the performance criteria for VCQA method any longer (ENGL, 2015). Based on these results, we speculated that the VCQA method allows the quantification and detection of level down to 1% and 0.1% of *Asarum sieboldii* Miq., which correspond to 1664.3 and 166.4 copy numbers, respectively.

### Application of Vector Control Quantitative Analysis Method to Commercial Herbal Formula Products

In order to demonstrate the practicability of VCQA method, 10 commercially available ChuanXiong ChaTiao Wan products randomly selected and purchased from different manufacturers were analyzed. The results indicated the quality of ChuanXiong ChaTiao Wan formula varies significantly among different manufacturers (Table 3). Generally, the proportion of quantified *Nepeta cataria* L. and *Mentha canadensis* L. was higher in relation to the other quantifiable species in most of the products. This case was expected as these two species, being the cheapest biological ingredients of all, would be added preferentially with larger amounts in the production of ChuanXiong ChaTiao Wan. Surprisingly, in one sample (COM_09), the percentage of *Asarum sieboldii* Miq. was unexpectedly almost 7% higher than the prescript of the Chinese Pharmacopoeia. In order to rule out that this result was not caused by artificial error, we reanalyzed this sample but found no significant deviation. A possible explanation for this perceived discrepancy may be a self-patent preparation of the product. Although no stringent stipulations require the manufacturer to list the species dosage in herbal medicinal product, a higher concentration of toxic component may pose a serious health issue to the end users. In addition, the quantitative results also reveal that another two products (COM_3 and COM_4) were much likely not containing any *Notopterygium incisum* K. C. Ting ex H. T. Chang since the presented Ct values were all lower than the limit of detection. After careful verification, we found that the price of these two products was almost a third lower than other products, which indicated that while *Notopterygium incisum* K. C. Ting ex H. T. Chang is the most expensive of all the tested species, the possibility exists for manufacturers to fraudulently increase the competitiveness of their products.

**TABLE 3:** Application of VCQA method for the quantitative determination of various commercial ChuanXiong ChaTiao Wan products.

Samples	Species content (mg/mg)							
	*Ligusticum sinense*	*Angelica dahurica*	*Notopterygium incisum*	*Asarum sieboldii*	*Saposhnikovia divaricata*	*Nepeta cataria*	*Mentha canadensis*	*Glycyrrhiza uralensis*
COM_1	0.1038 ± 0.0092	0.0517 ± 0.0047	0.0545 ± 0.0026	0.0258 ± 0.0016	0.0394 ± 0.0019	0.1259 ± 0.006	0.2420 ± 0.0113	0.0529 ± 0.0019
COM_2	0.1271 ± 0.0066	0.0629 ± 0.0041	0.0626 ± 0.0041	0.0297 ± 0.0008	0.0466 ± 0.0022	0.1418 ± 0.0101	0.2985 ± 0.0055	0.0679 ± 0.0022
COM_3	0.0956 ± 0.0048	0.0475 ± 0.006	—	0.0234 ± 0.0032	0.0363 ± 0.0014	0.1200 ± 0.0095	0.2411 ± 0.0063	0.0474 ± 0.0006
COM_4	0.1182 ± 0.0103	0.0591 ± 0.0074	—	0.0311 ± 0.005	0.0445 ± 0.0027	0.1435 ± 0.0092	0.3427 ± 0.0218	0.0590 ± 0.0018
COM_5	0.1388 ± 0.0035	0.0703 ± 0.0083	0.0485 ± 0.006	0.0318 ± 0.0028	0.0588 ± 0.005	0.1574 ± 0.0133	0.3166 ± 0.01	0.0711 ± 0.0076
COM_6	0.1376 ± 0.0061	0.0669 ± 0.0029	0.0601 ± 0.0018	0.0351 ± 0.0016	0.0509 ± 0.0006	0.1710 ± 0.002	0.2915 ± 0.0085	0.0614 ± 0.0008
COM_7	0.1020 ± 0.0073	0.0524 ± 0.0064	0.0450 ± 0.0033	0.0246 ± 0.0007	0.0369 ± 0.0013	0.1150 ± 0.0082	0.2306 ± 0.0093	0.0548 ± 0.0044
COM_8	0.1407 ± 0.0114	0.0700 ± 0.0099	0.0617 ± 0.0072	0.0362 ± 0.0055	0.0536 ± 0.0029	0.1594 ± 0.0125	0.3517 ± 0.0127	0.0774 ± 0.0082
COM_9	0.1156 ± 0.0088	0.0546 ± 0.0071	0.0517 ± 0.0047	0.0308 ± 0.0029	0.0463 ± 0.0018	0.1271 ± 0.008	0.2478 ± 0.0203	0.0577 ± 0.0045
COM_10	0.1447 ± 0.0109	0.0722 ± 0.0068	0.0718 ± 0.0051	0.0364 ± 0.0024	0.0532 ± 0.0006	0.1676 ± 0.0165	0.2883 ± 0.0074	0.0804 ± 0.0026

Note: values are the means of three replicate analyses.

### A Practical Research Flow Chart for Species Quantification in Herbal Formula Using Vector Control Quantitative Analysis

**FIGURE 6:**
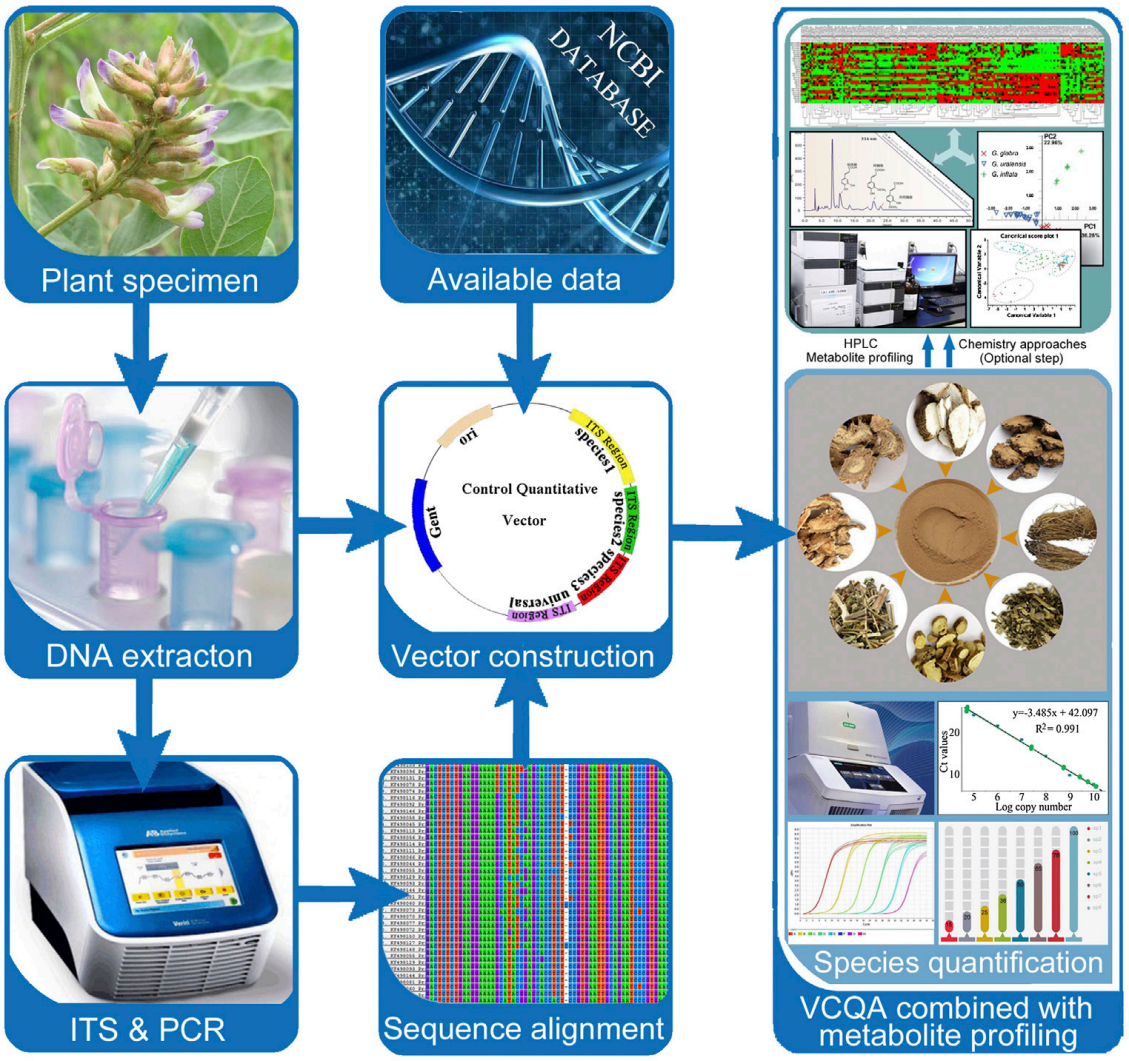
The research flow chart for species quantification in herbal formula using VCQA.

As the results described above, VCQA could be used to quantify the species composition in reference herbal formula and commercial products. Based on the protocol used in this study, we develop a practical research flow chart for species quantification in herbal formula using VCQA (Figure 6). First, genomic DNA from the original plant of each species is extracted respectively. The ITS sequence is then amplified by PCR using the extracted DNA. Second, ITS sequences from all the target species are aligned to find conserved sites for designing universal primers, as well as to identify species divergence sites for designing species-specific primers. Third, the species-specific primers are crosschecked among all the target species to ensure their specificity and amplification efficiency. Fourth, multiple target sequences are assembled into the pDONR207 vector to construct a quantitative plasmid by using a restriction–ligation–recombination reaction strategy. Fifth, a preliminary test is conducted to evaluate the quantified capacity of VCQA in reference experimental herbal formula and then apply it to quantify the species composition in commercial herbal formula products. Finally, if the results of tested commercial products deviate from the theoretical characterization of the official statement, metabolite profiling methods can be alternatively used to assist in metabolomic characterizing to verify and guarantee the VCQA results.

## Discussion

As highlighted by the recent authenticity survey of herbal medicines sold in China, Thailand, and North American, herbal medicine products are particularly vulnerable to commercial frauds (Newmaster et al., 2013; Osathanunkul et al., 2015b; Han et al., 2016). Although progress has been made in species-level authentication of most single herbs via DNA barcoding, species identification and quantification in complex herbal formulas are still rarely concerned. Although there are two reports that used Next Generation Sequencing (NGS) approach to identify and quantify species in herbal formulas (Cheng et al., 2014; Xin et al., 2018), it is difficult to widely apply it in herbal medicine detection mainly due to the sequence data and its analysis need highly skilled individuals and specialized bioinformatics programs. Moreover, although the cost per read for NGS approach is actually much lower than for Sanger sequencing, the price of equipment and consumables is not easily accessible for those starting out in molecular methods. Actually, most of these kinds of studies are conducted by external companies, which will lead to more time consumption. Thus, the development of more convenient and economical methods for the detection of species ingredient in complex herbal products is of great significance for herbal industry.

To meet such needs, a novel VCQA method is developed for quantifying multiple plant species in complex herbal product in this study. The backbone of our quantitative vector is based on the commonly used pDONR207 vector, which is suitable for inserting relatively long fragments (up to approximate 5 kb) by using Gateway recombination method. We used PCR-based strategy to quickly generate multiple species-specific fragments with designated adapters. Although many herbal formulas contain several plant species, the Golden Gate ligation combined with Gateway recombination techniques could simultaneously deliver multiple species-specific fragments (up to eight in this study) into the pDONR207 vector in a single cloning reaction (Figure 3). Notably, if the ligated multiple fragments were further processed by an additional PCR and then Gateway recombination was performed with the quantitative vector (see Methods), the efficiency of cloning can be significantly enhanced. Based on the comprehensive evaluation of eight target fragments in qPCR analysis, we found that VCQA method has an accurate quantifying capacity for ChuanXiong ChaTiao Wan formula. All the quantitative results present in this study showed high efficiencies of more than 90%, high *R* ^2^ values (*R* ^2^ ≥ 0.99), and very low standard deviation in each target fragment.

As we know, a big challenge in the quantitative evaluation of processed herbal product using DNA-based method is requiring a good quality of the initial DNA product against the backdrop of the diverse properties of medicinal parts and different production style of herbal medicine formulas. DNA isolated from different formula products was highly variable in quality and concentration. In order to address this issue, the universal primers and species-specific primers were introduced for the quantification. A major advantage of VCQA is that this method considers the factors affecting such as DNA degradation and inhibition when performing quantitative assessment. Without using universal primers, it would be difficult to determine whether variations in species-specific primer response were caused by the differences in species content or other factors such as DNA degradation or inhibition or differences in the amount of DNA added to the qPCR. By allowing both comparative measurements (Ct from species-specific against Ct from universal signal), the normalized calibration curves can be obtained. This process will reduce the inaccuracies caused by the uncertainty factors as described above.

Although the VCQA method has a potential power for biological ingredients quantification of herbal medicine formulas, there are still some limitations, such as the fact that some closely related species may not be quantified due to the limit sequence divergence in ITS region among these species, while current research advancement has not yet exploited optimal barcode markers that exhibit enough divergence for most closely related species. In addition, the accuracy of the VCQA method could be affected by DNA degradation and the presence of different plant tissue which potentially yielded different DNA amounts. More critical testing on VCQA using blind samples and different control samples is necessary to assess the accuracy of this technique. It is worth noting that the VCQA method is not suitable for quantifying the herbal medicine formulas which just contain plant extracts. We recommend combining the DNA molecular approaches for species quantification with analytical chemistry approaches for compounds determination to ensure the quality of herbal medicine formulas in a more thorough manner.

## Conclusion

VCQA method has proven to be a highly efficient, easy-to-use technique to quantify eight plant species in ChuanXiong ChaTiao Wan formula in this study. The fragment quantified using the ITS region had enough sequence divergence for designing species-specific primer, and it also had conserved sites for designing universal primer to amplify a short size fragment. By constructing a quantitative plasmid, absolute quantification of ChuanXiong ChaTiao Wan formula is presented. Most importantly, the species content is described in mg/mg, consistent with the norms described in the Chinese Pharmacopoeia. The VCQA method with its reliance on the ITS universal fragment and multiple quantification nature (i.e., multiple species are quantified in parallel relative to the universal fragment content) provides a unique alternative for the quantification of various herbal medicine formulas, especially the complex herbal formulas comprising multiple plant species.

## Data Availability Statement

The raw data supporting the conclusions of this article will be made available by the authors, without undue reservation, to any qualified researcher.

## Author Contributions

BZ, XD, and WS conceived and designed the experiments. CX and JL performed the experiments. BZ and CX analyzed the data. DZ and YS contributed reagents/materials/equipment. BZ and CX wrote the paper. XD and WS revised and approved the final version of the paper.

## Funding

This work was supported by Fund of National Natural Science Foundation of China (31960276) and Guangxi karst plant conservation and restoration ecology key laboratory construction project (17-259-23), and the Fundamental Research Funds for the Central public welfare research institutes (ZZ11-096).

## Conflict of Interest

The authors declare that the research was conducted in the absence of any commercial or financial relationships that could be construed as a potential conflict of interest.
