# Supplementary material for: Species Quantification in Complex Herbal Formulas—Vector Control Quantitative Analysis as a New Method
Source: Front Pharmacol. 2020 Nov 26;11:488193. doi: 10.3389/fphar.2020.488193 (PMC7725679; doi:10.3389/fphar.2020.488193)
Supplement: Supplementary file 1 [file table1.doc]

Supplementary Table S1. Primers used in this study.

| Primer name | Sequence (5’--3’) |
| --- | --- |
| ITS4 | TCCTCCGCTTATTGATATGC |
| ITS5 | GGAAGTAAAAGTCGTAACAAGG |
| Pps-LsITS_F | GGGGACAAGTTTGTACAAAAAAGCAGGCTTCCACACTCCTTCAAGAGCTGT |
| Pgs-LsITS_R | AGCGTGggtctcGtcagggTGCCTAGGGGTCCTAGAG |
| Pps-AdITS_F | TTCAGAggtctcTctgacacCAATTTCCGCCCCAAACCGG |
| Pgs-AdITS_R | AGCGTGggtctcGtcttcacGCACGTTAAAAATTTAGGTG |
| Pps-NiITS_F | TTCAGAggtctcTaagacttCCAGGAAGGTGGGYGCTCT |
| Pgs-NiITS_R | AGCGTGggtctcGagtccttCCAGACCGGCTCATCTCC |
| Pps-AsITS_F | TTCAGAggtctcTgactacaAACCCCGTCCCTTTGCAC |
| Pgs-AsITS_R | AGCGTGggtctcGgtccacaGTGGGAGCGACGCATAGC |
| Pps-SdITS_F | TTCAGAggtctcTggacttgTAGTTTCCGCCCCCAAACCTA |
| Pgs-SdITS_R | AGCGTGggtctcGcagatagACACGTCAACAATTTGGGCA |
| Pps-NcITS_F | TTCAGAggtctcTtctgcaaGGCTGTGAGGCACCGACA |
| Pgs-NcITS_R | AGCGTGggtctcGacctcaaCGTGCACAGCACGGTCGG |
| Pps-McITS_F | TTCAGAggtctcTaggtttcCGTTGGGTCGTTTTCGAT |
| Pgs-McITS_R | AGCGTGggtctcGagcgttcGGGATCGGGCGTCTATCA |
| Pps-GuITS_F | TTCAGAggtctcTcgctgatGCCACGCACTGTGTTCTC |
| Pgs-GuITS_R | GGGGACCACTTTGTACAAGAAAGCTGGGTCCCAACATTCACCCTGCAC |
| pDONR_F | TCGCGTTAACGCTAGCATGGATCTC |
| pDONR_R | GTAACATCAGAGATTTTGAGACAC |
| RT-LsITS_F | CACACTCCTTCAAGAGCTGT |
| RT-LsITS_R | TGCCTAGGGGTCCTAGAG |
| RT-AdITS_F | CAATTTCCGCCCCAAACCGG |
| RT-AdITS_R | GCACGTTAAAAATTTAGGTG |
| RT-NiITS_F | CCAGGAAGGTGGGYGCTCT |
| RT-NiITS_R | CCAGACCGGCTCATCTCC |
| RT-AsITS_F | AACCCCGTCCCTTTGCAC |
| RT-AsITS_R | GTGGGAGCGACGCATAGC |
| RT-SdITS_F | TAGTTTCCGCCCCCAAACCTA |
| RT-SdITS_R | ACACGTCAACAATTTGGGCA |
| RT-NcITS_F | GGCTGTGAGGCACCGACA |
| RT-NcITS_R | CGTGCACAGCACGGTCGG |
| RT-McITS_F | CGTTGGGTCGTTTTCGAT |
| RT-McITS_R | GGGGATCGGGCGTCTATCA |
| RT-GuITS_F | GCCACGCACTGTGTTCTC |
| RT-GuITS_R | CCAACATTCACCCTGCAC |
| RT-UNIV_F | TCGGGCGCAACTTGCGTTCA |
| RT-UNIV_R | GACTCTCGRCAACGGATATC |

**Note:** The green sequences in black box are introduced for Gateway BP recombination reaction. The underlined sequence is *Bsa*I site. The *Bsa*I-cutting ends with the same shaded color are compatible for ligation. The pink sequences are the species-specific primer sequences from the quantify plant species. The definition of primer names: LsITS is designed for *Ligusticum sinense*, AdITS is designed for *Angelica dahurica*, NiITS is designed for *Notopterygium incisum*, AsITS is designed for *Asarum sieboldii*, SdITS is designed for *Saposhnikovia divaricata*, NcITS is designed for *Nepeta cataria*, McITS is designed for *Mentha canadensis*, GuITS is designed for *Glycyrrhiza uralensis*.
