# Supplementary figures and images for: Species Quantification in Complex Herbal Formulas—Vector Control Quantitative Analysis as a New Method
Source: Front Pharmacol. 2020 Nov 26;11:488193. doi: 10.3389/fphar.2020.488193 (PMC7725679; doi:10.3389/fphar.2020.488193)

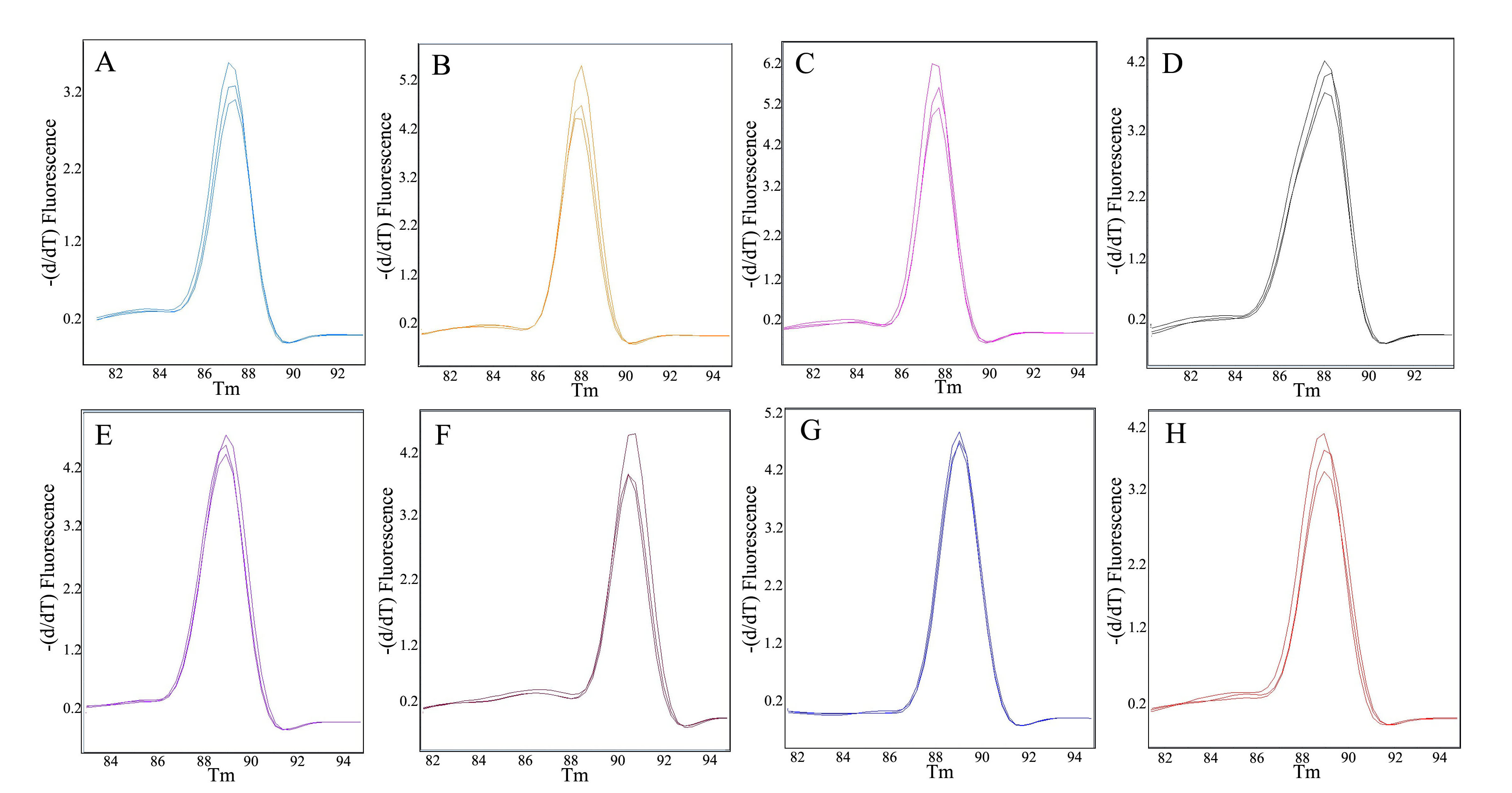

Supplement: Supplementary file 2 [file image1.jpeg]
